# The Impact of Polymer Size and Cleavability on the Intravenous Pharmacokinetics of PEG-Based Hyperbranched Polymers in Rats

**DOI:** 10.3390/nano10122452

**Published:** 2020-12-08

**Authors:** Nirmal Marasini, Changkui Fu, Nicholas L. Fletcher, Christopher Subasic, Gerald Er, Karine Mardon, Kristofer J. Thurecht, Andrew K. Whittaker, Lisa M. Kaminskas

**Affiliations:** 1 School of Biomedical Sciences, The University of Queensland, St Lucia 4072, Queensland, Australia; christopher.subasic@uq.net.au; 2 Australian Institute for Bioengineering and Nanotechnology, The University of Queensland, St Lucia 4072, Queensland, Australia; changkui.fu@uq.edu.au (C.F.); n.fletcher1@uq.edu.au (N.L.F.); tze.er@uq.net.au (G.E.); k.thurecht@uq.edu.au (K.J.T.); a.whittaker@uq.edu.au (A.K.W.); 3 ARC Centre of Excellence in Convergent Bio-Nano Science and Technology, The University of Queensland, St Lucia 4072, Queensland, Australia; 4 ARC Training Centre for innovation in Biomedical Imaging Technology, The University of Queensland, St Lucia 4072, Queensland, Australia; 5 Centre for Advance Imaging, The University of Queensland, St Lucia 4072, Queensland, Australia; k.mardon@uq.edu.au

**Keywords:** hyperbranched polymer, nanoparticles, nanomaterials, poly (ethylene glycol), pharmacokinetics, biodistribution, nanomedicine

## Abstract

A better understanding of the impact of molecular size and linkers is important for PEG-based hyperbranched polymers (HBPs) intended as tailored drug delivery vehicles. This study aimed to evaluate the effects of crosslinker chemistry (cleavable disulphide versus non-cleavable ethylene glycol methacrylate (EGDMA) linkers) and molecular weight within the expected size range for efficient renal elimination (22 vs. 48 kDa) on the intravenous pharmacokinetic and biodistribution properties of ^89^Zr-labelled HBPs in rats. All HBPs showed similar plasma pharmacokinetics over 72 h, despite differences in linker chemistry and size. A larger proportion of HBP with the cleavable linker was eliminated via the urine and faeces compared to a similar-sized HBP with the non-cleavable linker, while size had no impact on the proportion of the dose excreted. The higher molecular weight HBPs accumulated in organs of the mononuclear phagocyte system (liver and spleen) more avidly than the smaller HBP. These results suggest that HBPs within the 22 to 48 kDa size range show no differences in plasma pharmacokinetics, but distinct patterns of organ biodistribution and elimination are evident.

## 1. Introduction

Nanotechnology has evolved rapidly in modern medicine with a diverse range of materials in the clinic or under investigation for applications ranging from diagnostics and tissue-imaging agents, to cell and tissue delivery of drugs and genes [1,2]. The greatest hurdles to the successful translation of novel nanomedicines include chemical and biological reproducibility, delivery and toxicity issues, followed by the cost of synthesis [3,4,5]. For instance, in the context of tumour-targeted nanomedicines, a recent report showed that less than 1% of an intravenous nanomaterial dose reaches solid tumour tissue [6]. Furthermore, while nanomaterials based on highly biocompatible lipids and polymers, such as poly(ethylene glycol) (PEG), chitosan and phosphatidylcholine generally show good in vivo and in vitro safety profiles, they can exhibit varying degrees of immunogenicity and toxicity in certain organs [7,8,9,10,11]. These problems often arise due to a lack of comprehensive understanding of the in vivo fate of the nanomaterial, which can lead to suboptimal pharmacokinetics and biodistribution, and unpredictable toxicity profiles, particularly for large, long-circulating nanomaterials that are not readily cleared via the urine or faeces. While online tools exist to predict the pharmacokinetics, biodistribution and toxicity of small molecule drug candidates, and more recently dendrimers, based on decades of experimentation, at present, these properties, can only be identified for novel nanomaterials through in vivo experimentation [12,13,14]. It is therefore important to develop a thorough biopharmaceutical understanding of novel, biocompatible nanomaterial-based drug delivery platforms that have the significant potential to deliver drugs, imaging agents or genetic material payloads to target sites.

FDA-approved PEG is the most widely used polymer in biomedical applications due to its well-established safety profile [15]. PEG is hydrophilic and biocompatible and can be chemically conjugated, physically cross-linked or adsorbed onto the surface of nanoparticles. The presence of PEG imparts steric stabilisation onto nanoparticles by reducing charge-based interactions with proteins (stealth effect), thereby minimising clearance by macrophage-rich organs of the mononuclear phagocyte system (MPS) [16,17]. The pharmacokinetics of PEG-based polymers can generally be tailored by manipulating PEG loading and PEG molecular weight and branching [18,19]. For example, the influence of the overall molecular weight and PEG chain length has been characterised for dendrimers and star polymers [18,20]. However, extrapolation of these data to predict likely changes in the pharmacokinetics and biodistribution for other PEG-based polymers is not possible due to differences in polymeric architecture, chemical composition, shape, flexibility and hydrodynamic size.

Hyperbranched polymers (HBPs) are highly branched three-dimensional macromolecules that can have a dendrimer-like architecture. They possess multiple tuneable functional arms for the attachment of drugs and imaging agents without any entanglements [21]. In contrast to dendrimers that are prepared using complex synthetic and purification processes, HBPs are relatively easy to synthesise in a ‘single pot’ reaction at high yield. Synthesised HBPs also retain similar physicochemical properties to conventional dendrimers, but are considerably cheaper to synthesise, making them suitable for the mass production of cost-effective nanomedicines [22]. Some studies have examined the potential use of PEG-based HBPs as a nanocarrier for radioactive tracers (PET imaging), aptamers, genes and drugs [23,24]. However, these studies were primarily focused on elucidating the pharmacokinetics and biodistribution of constructs manufactured with a defined size and crosslinker chemistry. A comparison of the impact of molecular weight and crosslinker chemistry, particularly comparing cleavable and non-cleavable crosslinkers, on HBP pharmacokinetics and biodistribution has yet to be undertaken. The incorporation of cleavable crosslinkers is expected to allow HBPs to be more effectively eliminated from the body by disassembling the structures into smaller fragments triggered by specific chemical or biological stimuli, such as presence of glutathione (GSH), to prevent long-term in vivo retention and related toxicity. To this end, it is important to also define the impact of HBP size within the range expected, based on the dendrimer literature, to give constructs that exhibit prolonged plasma circulation, but efficient elimination via urine (approximately 20–50 kDa) [18,25].

The goal of this work was therefore to evaluate what impact a model cleavable crosslinker has on the pharmacokinetics and biodistribution of PEG-based HBPs of a given size and to evaluate how HBP size, within the range of approximately 20–50 kDa, affects pharmacokinetics. Two specific molecular weights (approximately 22 and 48 kDa) were chosen as they fall within the physiological threshold of urinary filtration [26]. Crosslinkers included cleavable disulphide-based dimethacrylate and non-cleavable ethylene glycol dimethacrylate (EGDMA), as these are readily incorporated into the molecular structure of the HBP. Three HBPs (non-cleavable 22 kDa, plus cleavable and non-cleavable HBPs of approximately 46–48 kDa) were synthesised and labelled with a radioactive tracer (^89^Zr) to allow positron emission tomography (PET) imaging of HBP biodistribution over time in concert with serial sampling of blood to quantify plasma pharmacokinetics via gamma counting.

## 2. Materials and Methods

### 2.1. Materials

Poly(ethylene glycol) methyl ether methacrylate (PEGMA) (MW 480), 4-cyano-4-((dodecylsulfanylthiocarbonyl)sulfanyl)pentanol, ethylene glycol dimethacrylate (EGDMA), 2-aminoethyl methacrylate hydrochloride (AEMA), 2,2′-azobis(2-methylpropionitrile) (AIBN), *N*,*N*′-bis(acryloyl)cystamine (BAC) were purchased from Sigma Aldrich (Castle Hill, Australia). Chelator p-SCN-Bn-Deferoxamine (DFO) was purchased from Macrocyclic Inc. (Plano, IL, USA). The inhibitors were removed from monomers by passing through a short basic aluminium oxide column. ^89^Zr oxalate was supplied by Perkin Elmer (Melbourne, Australia). All other chemical reagents were ARC grade and used as received from the suppliers.

### 2.2. Methods

#### 2.2.1. Synthesis of Hyperbranched Polymers

HBPs were synthesised using slightly modified procedures to those reported previously using the reversible addition-fragmentation chain transfer (RAFT) polymerisation technique [23,27,28]. The materials and quantities used are listed in Table S1 (supporting information). In a typical procedure, functional monomer (PEGMA) and amino-monomer (AEMA), RAFT agent (4-cyano-4-([dodecylsulfanylthiocarbonyl] sulfanyl) pentanol), crosslinking monomers (EGDMA or BAC) and free radical initiator (AIBN) were dissolved in DMF (5 mL) in the polymerisation flask. The flask was hermetically sealed with a rubber septum, and the solution was purged with a gentle flow of argon for 20 min. The polymerisation was carried out in an oil bath at 70 °C under magnetic stirring for 72 h. The polymerisation was quenched by exposing the reaction vessel to air. The polymerisation led to at least 95 mole percent of full monomer PEGMA conversion as determined from ^1^H NMR. The polymers were purified by precipitating into cold diethyl ether followed by centrifugation at 2000× *g* for 5 min to obtain crude brown oily polymers. The polymers were further vacuum-dried overnight at room temperature.

The amine functionalised HBPs were further reacted with the chelator deferoxamine (DFO) to yield DFO-conjugated HBP. In brief, polymers (1 µM) dissolved in 1.5 mL of 0.1 M aqueous solution of sodium carbonate and DFO (6 µM; dissolved in 450 µL of DMSO) were mixed together in a 5 mL round bottom flask. The solutions were reacted under magnetic stirring at room temperature for 24 h. DFO-conjugated HBPs were purified by removing free DFO by dialysis (MW cut off: 3500 Da) against 2 litres of milli Q water for at least 72 h with 6–12 hourly changes of water. The resultant DFO-conjugated polymers were further purified using Hi-trap desalting columns (MWCO 7K, Thermo Fisher Scientific, Brisbane, Australia) to remove unreacted DFO according to the manufacturer’s protocol. The purified products were analysed by ^1^H NMR, size exclusion chromatography (SEC) and particle size via dynamic light scattering (DLS), as described in the supporting information prior to subsequent labelling with ^89^Zr. The final size and nomenclature for the synthesised (unlabelled) HBPs are listed in Table 1.

^89^Zr oxalate dissolved in 1 M Oxalic acid (Perkin Elmer) was neutralised with 1 M Na_2_CO_3_ (1:0.82, ^89^Zr oxalate: 1 M Na_2_CO_3_) and added to an equivalent volume of 0.5 M HEPES pH 7.5 buffer. DFO functionalised NC-HBP-22K (4.98 mg), NC-HBP-48K (11.25 mg) and C-HBP-46K (18.46 mg) in 0.5 M HEPES buffer were added to aliquots of buffered ^89^Zr (NC-HBP-22K; 108.0 MBq, NC-HBP-48K; 116.5 MBq, C-HBP-46K; 100.7 MBq) and labelling was allowed to proceed at 37 °C for 1 h with shaking at 400 rpm. Samples were buffer exchanged into 50 mM sterile phosphate-buffered saline (pH 7.4) using Zeba Spin Desalting Columns (7 kDa MWCO, Thermo Fisher Scientific). To assess the presence of free ^89^Zr or small molecule fragments, 1 µL samples of each solution were taken and combined with 1 µL 50 mM diethylenetriaminepentaacetic acid (DTPA). These were then spotted on thin layer chromatography paper (Agilent iTLC-SG Glass microfiber chromatography paper impregnated with silica gel) and run using 50:50 H_2_O: EtOH as an eluent. The elution behaviour of unbound neat ^89^Zr and DTPA complexed ^89^Zr was used to compare with the polymer samples. Plates were then dried and imaged on a Bruker In Vivo MS FX Pro imaging system using a radioisotopic phosphor screen. All samples with radiolabelling purities ≥95% were used for in vivo experiments. All samples were then sterile filtered (0.22 µm Cathivex-GV sterile filter (Merck Millipore, Bayswater, Australia)) before injection into rats. The final radiolabel concentration of each polymer was 7.4, 3.9 and 1.8 MBq/mg for NC-HBP-22K, NC-HBP-48K and C-HBP-46K, respectively.

#### 2.2.2. Cell Culture and Cytotoxicity Assay

MDA-MB-231 human breast adenocarcinoma cells (obtained from ECACC, Sigma and tested negative for mycoplasma) were grown in RPMI (Sigma-Aldrich, Castle Hill, Australia) supplemented with 10% fetal bovine serum and penicillin (100 IU/mL) and streptomycin (100 ng/mL) at 37 °C and 5% CO_2_. Cells were plated at a density of 1 × 10^4^ cells^/^well in a 96-well plate in a final volume of 100 µL supplemented media. After overnight adherence, the cell culture medium was exchanged with a serum-free media containing supplements, as described previously [29]. Cells were treated with either medium alone or polymers at concentrations at or below 4 mg/mL for 72 h. Cell density was then evaluated via 3-(4,5-Dimethylthiazol-2-yl)-2,5-diphenyltetrazolium bromide (MTT) assay (Sigma Aldrich, Castle Hill, Australia) by adding 10 µL of a 10 mg/mL solution per well, as described previously [30]. The cells were incubated at 37 °C for a further 3 h, and the supernatant then removed and replaced with DMSO (100 µL/well) to dissolve the formazan crystals. Cell viability was then evaluated using a microplate reader by measuring absorbance at 540 nm for the treated cells versus untreated control.

#### 2.2.3. Animals

All animal care and experimental procedures were carried out with the approval of the Animal Ethics Committee of The University of Queensland and following the guiding principles of the Australian Code for the Care and Use of Animals for Scientific Purposes (SBS/256/NHMRC/UQ)**.** Nine male Sprague-Dawley rats (5–7 weeks, three per group) were purchased from the Animal Resource Centre (Perth, Australia) and acclimatised for 2 weeks prior to surgery. Animals were always maintained on a 12 h light/dark cycle and provided with free access to water. Food was always provided except after surgery (until 4 h post dose after PET imaging).

#### 2.2.4. Surgical Cannulation of the Carotid Artery and Jugular Vein of Rats

Animals were transferred to individual metabolism cages the day before surgery and housed in the same cage throughout the study to allow separate collection of urine and faeces. Rats were anesthetised using 2–3% isoflurane in O_2_ for surgical cannulation and were provided Carprofen (8 mg/kg, s.c) and Marcaine (0.5%, 50 µL per incision site) for post-surgical pain relief. Polyethylene cannulas (0.96 mm external diameter, 0.58 mm internal diameter, Microtube Extrusions, North Rocks, Australia) were inserted into the right carotid artery and jugular vein for blood sampling and drug dosing, respectively, as described previously [30]. Following surgical implantation of cannulas, rats were attached to harness and swivel assemblies to allow serial blood sampling from freely moving rats as previously described [31,32]. Rats were allowed to recover overnight prior to dosing.

#### 2.2.5. Pharmacokinetic and Biodistribution Analysis of HBPs after Intravenous Administration to Rats

Rats were divided into three groups of three rats. Pre-dose blood sample (200 µL) were initially collected from the carotid artery cannula into heparinised Eppendorf tubes (10 IU heparin). Rats were weighed prior to dosing. They were then administered ^89^Zr-labelled HBPs over 2 min as an intravenous dose in a 1 mL final volume of sterile saline via the jugular vein cannula. Rats were dosed with sufficient polymer to give reliable PET images (equivalent to 10–20 mg/kg polymer, or 7.5–14.5 MBq ^89^Zr) in approximately 500–600 µL of saline (Baxter Healthcare Ltd., Victoria, Australia), which was followed by infusion of a further 400–500 µL of saline to flush through any dose remaining in the jugular vein cannulas. After dosing, additional blood samples (200–250 uL) were collected immediately after the completion of dosing (time 0), and at 1, 2, 4.5, 6, 8, 24.5, 48, and 72 h. Whole blood was centrifuged at 3500× *g* for 5 min to obtain plasma, and a 100 µL aliquot was analysed for gamma radiation in a Perkin-Elmer Wizard gamma counter (PerkinElmer, Waltham, MA, USA). In addition, whole urine and faeces excreted each day were collected, and total radioactivity similarly quantified to determine proportion of the dose excreted via the urine and faeces. After the last sample was collected 72 h after dosing, rats were euthanised and liver, spleen, heart, lungs and kidneys removed, weighed and counted in 20 mL scintillation vials in the gamma counter.

#### 2.2.6. PET/CT Imaging of Rats for Time Course Evaluation of Biodistribution

At times 4, 24 and 72.5 h after dosing, rats were anaesthetised under isoflurane at a dose of 3% in a closed anaesthetic induction chamber and placed in the PET/CT small animal imager (Inveon preclinical multimodality PET/CT scanner; Siemens, Erlangen, Germany). Rats were monitored using ocular and pedal reflexes to ensure deep anaesthesia outside of the imager. Physiological monitoring inside the imager (breathing parameters using a sensor probe) was achieved during imaging using an animal monitoring system (BioVet^TM^ system, m2m Imaging, Salisbury, Australia). Static PET data acquisition was performed for approximately 30 min for each rat. The resulting data were reconstructed with a two-dimensional ordered-subset expectation maximisation (OSEM2D) algorithm using the Inveon Acquisition Workstation software (IAW version 4.1, Siemens). Following the PET scans, the CT images of the rats were acquired through an X-ray source with the voltage set to 80 kV and the current set to 500 µA. The scans were performed using 360° rotation with 120 rotation steps with a low magnification and a binning factor of four. The exposure time was 240 ms, with an effective pixel size of 106 µm. The entire CT scanning process took approximately 12 min. The CT images were reconstructed using Feldkamp reconstruction software (Siemens, Erlangen, Germany). CT and PET datasets of each individual animal were aligned using Inveon Research Workstation (IRW version 2.1, Siemens, Erlangen, Germany) to ensure good overlap of the organs of interest. Three-dimensional ROIs were placed within the whole body, as well as all the organs of interest, including the heart, kidneys, liver, gastrointestinal tract, knee joints and spleen using morphologic CT information to delineate organs. Activity per voxel was converted to nci/cc using a conversion factor obtained by scanning a cylindrical phantom filled with a known activity of ^89^Zr to account for PET scanner efficiency. Activity concentrations were then expressed as a percent of the decay-corrected injected activity per cm^3^ of tissue that can be approximated as percentage injected dose/gram (%ID/g). Activity concentrations were also normalised to the injected dose and the animal weight and expressed in standardised uptake value per body weight (SUV-BW).

#### 2.2.7. Size Exclusion Chromatography of Plasma and Urine

Plasma samples collected from rats at 0, 24, and 72 h, and urine collected over 0–24 h and 48–72 h were pooled after gamma analysis (within dose groups) and analysed via size exclusion chromatography (SEC) to identify whether ^89^Zr quantified in the samples was due to free radiolabel, high molecular weight material (>10 kDa) or intermediate sized (<10 kDa) material in these samples. This gave some information regarding whether HBP was a quantified or lower molecular weight material that may be liberated radiolabel or products of HBP degradation in vivo. Samples of free radiolabel and HBP were also analysed to identify elution volumes of these materials.

In brief, plasma (200 µL) or urine (500 µL) were separately added to Sephadex^TM^ G-25 columns (GE Healthcare, Parramatta, Australia) and eluted with phosphate buffer saline (12 mL). The eluant was manually collected in 0.5 mL fractions into 24 tubes and analysed for radioactivity on a gamma counter. Columns were washed with 24 mL water between runs to remove any remaining radiolabel and ensure that the same column could be used to separate each sample for a given HBP. This ensured that retention volumes did not change between columns for a given HBP. Manual Sephadex G25 columns were employed here, rather than Superdex columns linked to an HPLC as described by us previously [18,20], because biological samples containing gamma radiation were not permitted on the available HPLCs.

#### 2.2.8. Pharmacokinetic Calculations and Statistical Analysis

The concentration of HBPs in plasma samples measured by gamma counter were determined after decay correction of the samples to the time of injections using the following formula:

$$DPM=\left(\frac{CPM}{exp(-0.693*\left(\frac{\;time\;difference\;}{78.4}\right)}\right)/0.12$$

where *CPM* = counts per minute; *DPM* = disintegration per minute; *time difference* resembles the time difference between gamma counting and injection; 78.4 is half-life of ^89^Zr and 0.12 is efficiency constant to convert from CPM to DPM using the gamma counter. The DPM values were converted to megabecquerel (MBq) and calculated for the percentage of injected dose of the radioactivity (%ID) and amount of polymer (ug/mL) content using the specific activity of the ^89^Zr-labelled HBPs.

Pharmacokinetic parameters were manually calculated based on these concentration data. It is important to note that this approach assumes that polymers remained intact and associated with ^89^Zr in plasma. The terminal elimination rate constant (k_el_) was calculated by regression analysis of the terminal linear phase of the plasma concentration-time profile. Elimination half-life (*t*_1/2_) was calculated by dividing Ln2 by the k_el_ value. The linear trapezoidal rule was used to calculate the area under the plasma concentration-time curve (AUC_0–72 h_). AUC after the last sample and extrapolated to infinity (AUC_72–∞_) was calculated by dividing the plasma concentration at last timepoint by k_el_. The initial volume of distribution (*V*_c_) was calculated by dividing the dose administered by the concentration of drug at *t = 0* and the terminal volume of distribution (VD_β_) was calculated by dividing the dose administered by AUC_0–∞_ and multiplied by k_el_. Clearance (Cl) was determined by dividing the administered dose by AUC_0–∞_. Pharmacokinetic parameters and organ biodistribution among polymers were compared using one-way analysis of variance (ANOVA) followed by Tukey’s test for the statistical significance. Significance was determined at *p* < 0.05.

## 3. Results

### 3.1. Chemical and Cell-Based Characterisation of Hyperbranched Polymers

**Figure 1:**
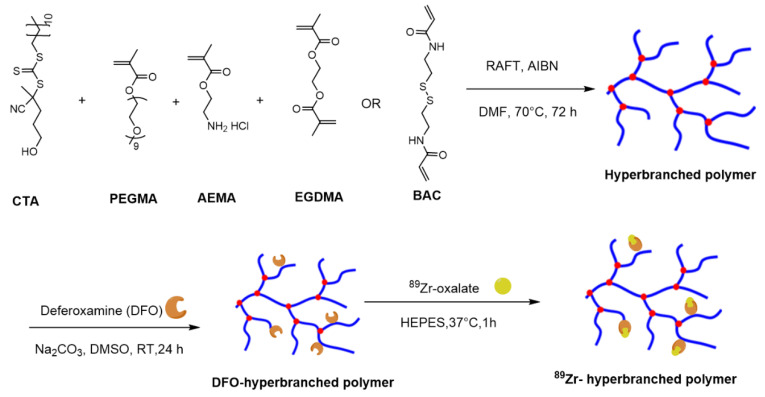
Schematic representation of typical approaches to synthesise non-cleavable and cleavable PEG-based hyperbranched polymers and subsequent steps to conjugate chelator, deferoxamine and ^89^Zr. NC-HBP-22K and NC-HBP-48K employed a non-biodegradable ethylene glycol dimethacrylate crosslinker, while C-HBP-46K employed a biodegradable N, N′-Bis(acryloyl)cystamine (BAC) functionality as the crosslinker. CTA (chain transfer agent): 4-cyano-4-[(dodecylsulfanylthiocarbonyl)sulfanyl]pentanol; PEGMA: Poly(ethylene glycol) methyl ether methacrylate (PEGMA); EGDMA: ethylene glycol dimethacrylate; AEMA: 2-aminoethyl methacrylate hydrochloride), BAC: 2,2′-azobis(2-methylpropionitrile); AIBN: *N*,*N*′-bis(acryloyl)cystamine (BAC); RAFT: Reversible addition-fragmentation chain-transfer.

HBPs with varying molecular weights and crosslinker chemistry were prepared using well-established protocols that utilise the RAFT technique. Typical synthetic steps used in the synthesis of HBPs are illustrated in Figure 1. In brief, three amino-functionalised HBPs were synthesised with a high degree of PEGMA conversion (≥95%) (Supplementary Information, Table S1). The detailed physiochemical characteristics of the synthesised HBPs are presented in Table 1. SEC, coupled with an RI detector, showed that the molecular weights of HBP-NC-22K, HBP-NC-48K and HBP-C-46K were approximately 22.3, 48.2 and 45.5 kDa, respectively. The molecular weight distribution (Đ) which was related to the degree of branching, a typical characteristic for HBPs, was between 1.7 and 2.1.

The ability to form unimolecular micelles was observed by dispersing HBPs (10 mg/mL) into milli Q water at room temperature (Table 1). The differences in polymer composition and molecular weight had an impact on the hydrodynamic diameter of the micelles. The smallest non-cleavable polymer (NC-HBP-22K) formed particles with a diameter of approximately 6 nm. In contrast, the hydrodynamic diameter of higher molecular weight cleavable and non-cleavable polymers were 8 and 9 nm, respectively. The zeta potential for the HBPs showed that they were mostly uncharged (Table 1). The HBPs were further conjugated to the chelator deferoxamine (DFO) (Supplementary Information Figure S1) and then radiolabelled with ^89^Zr.

**Figure 2:**
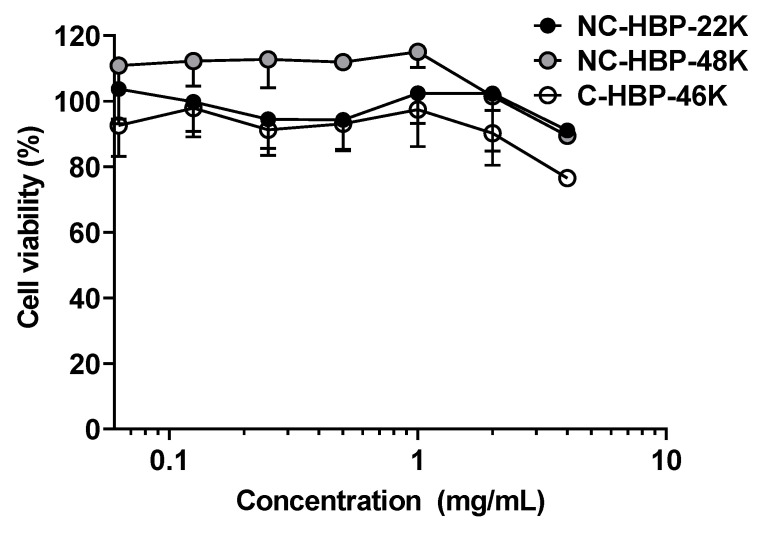
Cytotoxicity profile of hyperbranched polymers at various concentrations against MDA-MB-231 cells over a 72 h incubation period. Values are represented as mean ± SD (*n* = 3).

The in vitro cytotoxicity of the polymers were also examined against MDA-MB-231 breast cancer cells, as a model cell type, and were found to be non-toxic at concentrations below 2 mg/mL over 3 days, but inhibited cell growth by approximately 20% at 4 mg/mL (Figure 2).

### 3.2. Plasma Pharmacokinetics and Excretion of HBPs in Rats

**Figure 3:**
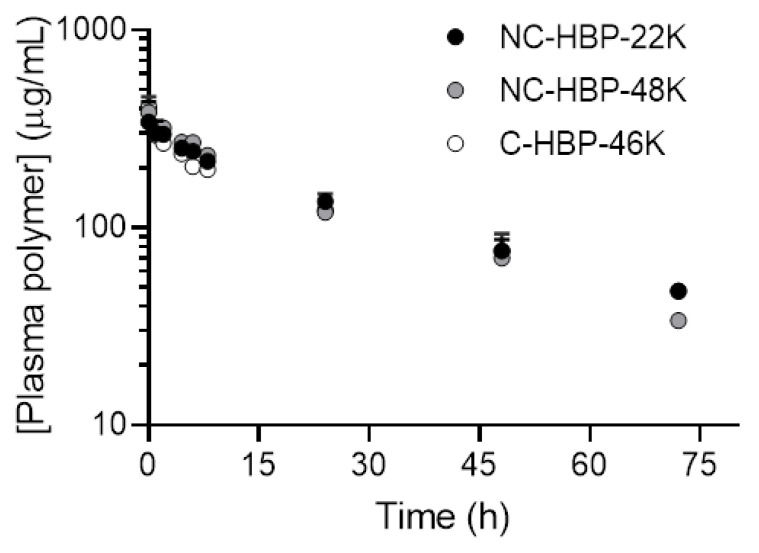
Plasma concentration versus time profile of ^89^Zr-conjugated HBPs after intravenous administration in rats. Plasma concentrations were normalised to a dose of 15 mg/kg to allow direct comparison of the plasma profiles for each polymer. Data are shown as mean ± SD (*n* = 3 rats, except NC-HBP-48K where *n* = 2 rats and data are represented as mean ± range).

The plasma concentration-time profiles of radiolabelled HBPs are shown in Figure 3, and pharmacokinetic parameters are reported in Table 2. The mean plasma concentration-time profiles for all HBPs were almost identical to each other over 72 h (Figure 3). With the exception of NC-HBP-48K, data represent mean and standard deviation from three animals. However, data from only two animals are reported for NC-HBP-48K, since some of the IV dose entered the subcutaneous space around the jugular vein cannula and showed a partial absorption profile, requiring elimination from the dataset. Given the short half-life of ^89^Zr (78.4 h), it was not feasible to cannulate and dose an additional rat to increase the n value, so mean and range are reported for NC-HBP-48K.

**Figure 4:**
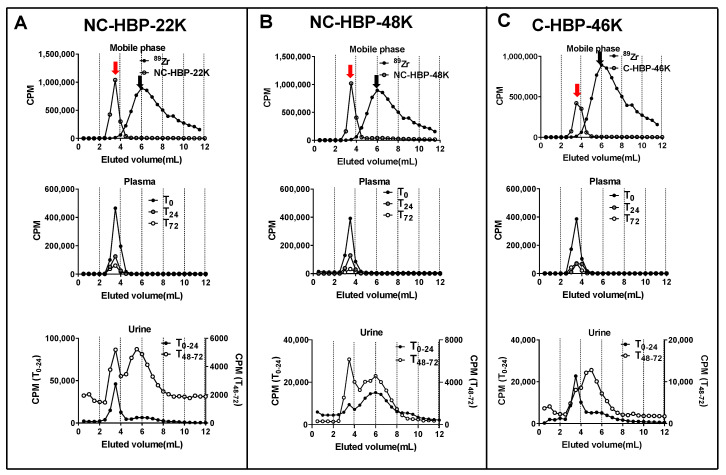
Size exclusion chromatography profiles of plasma and urine obtained from rats dosed intravenously with NC-HBP-22K, NC-HBP-48K, and C-HBP-46K. (**A**) Chromatogram of ^89^Zr (peak represented by black arrow) and NC-HBP-22K (peak represented by a red arrow) in mobile phase (top row), plasma (T_0_, T_24_ and T_72_ h) (middle row) and urine collected over time 0–24 h (T_0–24_) and 48–72 h (T_48–72_) (bottom row). (**B**) Chromatogram of ^89^Zr (peak represented by a black arrow) and NC-HBP-48K (peak represented by a red arrow) in a mobile phase (top row), plasma (T_0_, T_24_ and T_72_ h) (middle row) and urine collected over time 0–24 h (T_0–24_) and 48–72 h (T_48–72_) (bottom row). Panel (**C**) Chromatogram of ^89^Zr (peak represented by black arrow) and C-HBP-46K (peak represented by a red arrow) in a mobile phase (top row), plasma (T_0_, T_24_ and T_72_ h) (middle row) and urine collected over time 0–24 h (T_0–24_) and 48–72 h (T_48–72_) (bottom row). Both plasma and urine samples were pooled from all rats in the group for SEC and radioactivity measurements.

In general, the plasma pharmacokinetics between the three HBPs were very similar, with terminal half-lives ranging from approximately 25 to 35 h. Approximately 90% of the IV dose of each polymer was cleared from plasma over 3 days. The cleavable C-HBP-46K HBP showed a significantly lower V_c_ (approximately 7.3 mL) and higher elimination half-life (approximately 35 h) than the non-cleavable HBPs (V_c_ approximately 11.5 mL, half-life approximately 25 h), but overall plasma clearance did not differ significantly. The SEC profiles of ^89^Zr in plasma samples (Figure 4) indicated that all of the radiolabel quantified in plasma over 3 days was associated with high molecular weight (>10 kDa) material.

The proportion of the ^89^Zr dose recovered in the urine and faeces are presented in Table 2. Interestingly, despite the smaller size, NC-HBP-22K showed a similar excretion profile to the larger NC-HBP-48K, with minimal overall excretion over 3 days (approximately 2–4% dose in faeces and 6–9% dose in urine). SEC profiles did, however, show that the smaller HBP was more readily excreted unchanged in the urine, as evidenced by a more prominent peak corresponding to high molecular weight material in the 0–24 h and 48–72 h urine samples (Figure 4). In contrast, ^89^Zr associated with NC-HBP-48K was mainly eliminated as the free ^89^Zr or smaller fragments, suggesting that the larger non-cleavable polymer is not readily eliminated from the body. However, as expected, the high molecular weight cleavable HBP (C-HBP-46K) was excreted more readily via the urine and faeces (approximately 15 and 10%, respectively) than the non-cleavable polymers, despite the similar plasma pharmacokinetic profiles. The SEC profiles in urine also revealed the presence of material with a higher molecular weight than ^89^Zr (eluting between 3–5 mL, in contrast to 6 mL for free ^89^Zr) and a relative decrease in the proportion of larger molecular weight material at 3 mL, suggesting that products of HBP degradation were the predominant species excreted via urine for this polymer.

### 3.3. Organ Biodistribution of HBPs

**Figure 5:**
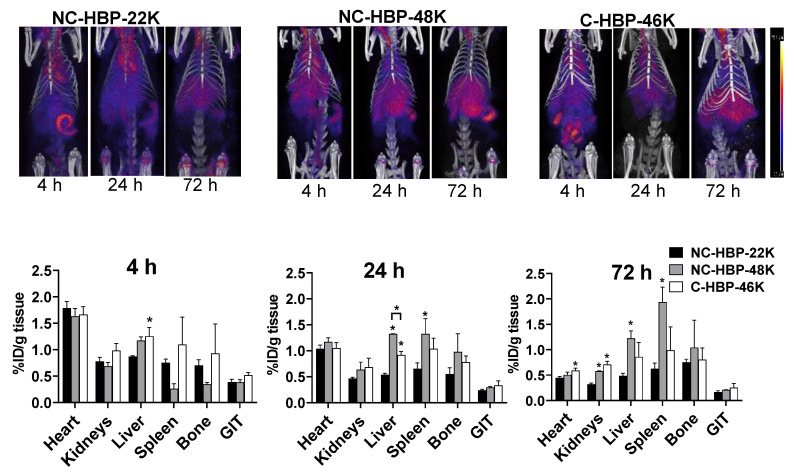
Representative PET/CT images of ^89^Zr-labelled HBPs acquired at 4 h, 24 h and 72 h post-injection for each HBP (top panels) and estimated Comparative biodistribution of HBPs at a various time interval in different organs (bottom panels). Region of interest (ROI) analysis of whole body and organs allow determination of in vivo distribution of HBPs in the organs. Biodistribution across organs at various timepoints were calculated by ROI analysis of PET/CT images. Images are decay corrected to the point of dosing and intensity in each organ is expressed as % injected dose/g of tissue (*n* = 3 rats for all groups except NC-HBP-48K, where *n* = 2 rats and data are represented as mean ± range). Statistical significance was conducted using one-way ANOVA followed by Tukey’s test. * indicates *p* < 0.05 compared with NC-HBP-22K. ID: Injected dose.

To evaluate the biodistribution of the polymers over time, rats were imaged in a PET/CT scanner at 4, 24 and 72 h. Polymer biodistribution was examined in the heart (as a representation of proportion of the injected dose in blood), liver, spleen, kidneys, bone and gastrointestinal tract (GIT). Representative images are shown in the top panels of Figure 5. Bottom panels show the organ biodistribution at each imaging timepoint (also represented as biodistribution over time for each polymer in the supporting information for reference). In general, PET images revealed a steady decline in heart, kidney and GIT distribution of the polymers over time, but relatively consistent levels in MPS organs (liver, spleen, and bone). The exception, however, was the distribution of NC-HBP-48K in spleen and bone, which showed a gradual accumulation of the dose over 3 days, reaching levels up to two-fold higher than the other polymers. The smallest NC-HBP-22K showed overall lower levels of organ retention compared to the larger polymers, but consistent with plasma pharmacokinetic data, heart distribution showed similar blood retention/exposure for all polymers, regardless of linker chemistry and size.

**Figure 6:**
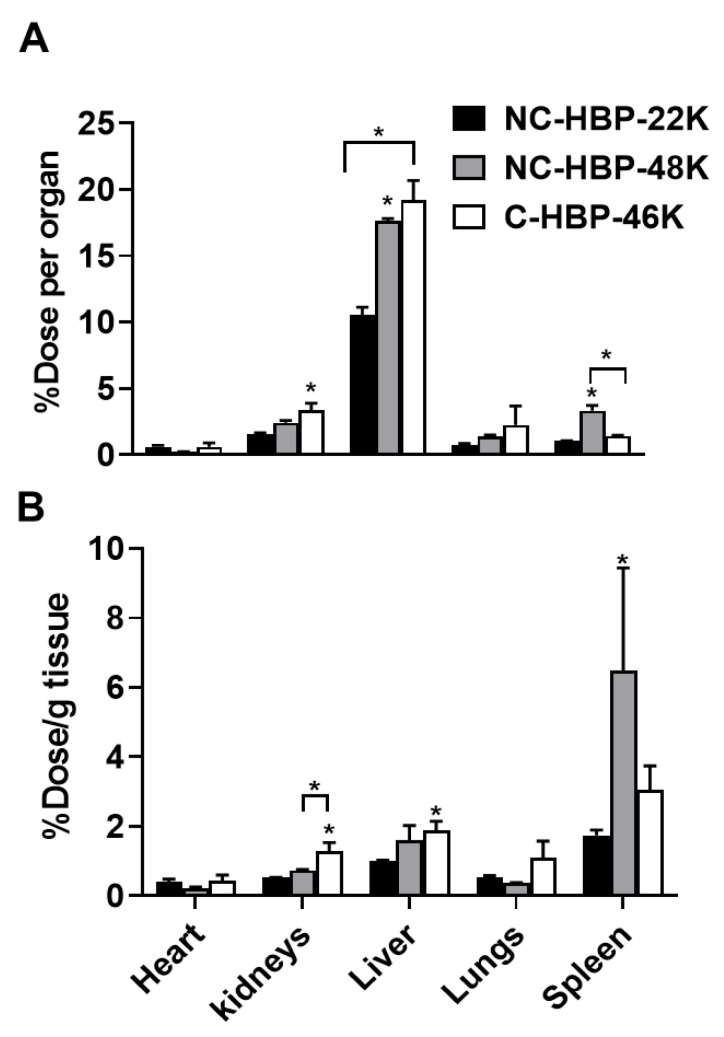
Organ biodistribution of ^89^Zr-labelled HBPs after intravenous administration in rats measured by gamma counting of extracted organs after termination. Panel (**A**) Percent of ^89^Zr dose recovered per organ. Panel (**B**) Percent of ^89^Zr recovered per gram of tissue. (*n* = 3 rats for all groups except NC-HBP-48K where *n* = 2 rats and data are represented as mean ± range). Statistical significance was conducted using one-way ANOVA followed by Tukey’s test. * indicates *p* < 0.05 compared with NC-HBP-22K.

While PET images can be used to estimate percent injected dose per gram of tissue (as shown in Figure 5), this approach is largely computational and not as accurate as quantifying the radiolabel content of extracted samples, and considerably less accurate for quantifying radiolabelled materials in lungs, where signal from the heart can cause interference. At the completion of blood sampling and imaging, rats were therefore culled, and major organs harvested to quantify ^89^Zr biodistribution via gamma counting. The percent of radioactivity dosed detected in each organ and per gram of tissue after correction for the weight of the extracted organ are presented in Figure 6A,B, respectively. In general, the pattern of biodistribution for the HBPs at 72 h matched the PET imaging data. These showed lower organ retention for the smallest polymer, and high spleen retention for NC-HBP-48K. The proportion of dose recovered per gram of tissue was similar to the PET-calculated data, but largely underestimated via PET imaging for some organs, in particular the spleen, which showed up to 8% dose/g for NC-HBP-48K.

Per whole organ, the liver and spleen were the primary target organs for HBPs (Figure 6). The distribution pattern of HBPs in the liver and spleen were found to be dependent on molecular weight, whereby approximately 20% of the larger HBPs were taken up by the liver (irrespective of the crosslinker) compared to 10% for the smaller HBP. Consistent with PET data, a significant proportion of NC-HBP-48K was also found in the spleen at 72 h (approximately 2-fold higher than the other HBPs) over time compared to NC-HBP-22K- and C-HBP-46K-dosed rats. The radioactivity of larger molecular weight HBPs (46 and 48 kDa) recovered in the liver and spleen were significantly higher than smaller molecular weight NC-HBP-22K. Kidney data also confirmed the urine excretion data presented in Table 2, since C-HBP-46K showed approximately 2-fold higher kidney retention compared to the non-cleavable constructs.

## 4. Discussion

Defined pharmacokinetic studies on PEG-based HBPs are usually limited to the understanding of biopharmaceutical behaviour of polymers with specific physicochemical properties and lack head-to-head comparisons with constructs generated with different crosslinkers and molecular weights, particularly over the size range explored here. The choice of crosslinker (cleavable and non-cleavable) in particular can dictate the patterns of polymer degradation and potentially accessibility of enzymes to peptide-cleavable drug linkers [33]. It can also dictate elimination pathways and rates from blood. Here, we investigated how changes in molecular weight and crosslinker chemistry (cleavable vs. non-cleavable) influence the biopharmaceutical behaviour of HBPs after intravenous administration in rats. PEGMA was used as a major repeating unit in the polymeric structure, and its inclusion in the hyperbranched structure improved the aqueous solubility of HBPs, which is essential when designing HBPs to carry hydrophobic drugs. We incorporated either relatively stable EGDMA or cleavable disulphide-based BAC as a crosslinker to bridge the polymer chains. AMA was included in the polymer design to provide post-polymerization modification sites to incorporate the radiometal chelator DFO. The materials were labelled with the long-lived positron-emitting isotope ^89^Zr that has a half-life of 78.4 h, allowing quantitative and sensitive analysis of pharmacokinetic and biodistribution profiles using PET imaging and ex vivo gamma scintillation counting.

HBPs formed unimolecular micelles of size <10 nm diameter and zeta potential values were either slightly negative or neutral in charge. The near-zero zeta potential of the HBPs is expected to reduce the interaction with opsonins (serum proteins) that promote targeting to MPS organs and prolong the circulation time of HBPs in vivo. It is widely recognised that biologically inert nanomaterials are generally safe and cytotoxicity is critically dependent on the charge, size and PEG density on the surface of particles [34,35]. Previous work has shown that nanomaterials bearing a neutral charge are generally safer than those with cationic or anionic charges [36,37]. The HBPs were investigated for cytotoxicity in MDA-MB-231 cells using a standard MTT assay to provide preliminary insights into the safety of our HBPs. It is noteworthy that neither molecular weight nor crosslinker chemistry affected cell viability. HBPs were found to be well tolerated by cells up to concentrations of 2 mg/mL (over 4-fold higher than the Cp_0_). Cell viability was reduced by approximately 20%, however, at a concentration of 4 mg/mL. This finding is consistent with previously published safety studies on PEG-based HBPs. For instance, a toxicity study in RAW 264.7 and 3T3 fibroblasts cells lines with poly (PEGMA)-based HBP showed more than 80% cell viability at 2 mg/mL [38]. Another study showed PEG-based HBPs exhibited at least 90% cell viability at a concentration up to 5 mg/mL against MDA-MB-231 cells [38]. Additionally, other studies found that PEG-based star polymers with cationic cores showed more than 80% cellular viability at a concentration of 0.8 mg/mL [39].

All HBPs showed similar plasma pharmacokinetics, with terminal half-lives of around from 25 to 35 h, regardless of size and the different crosslinkers used. This was in contrast to the effect of molecular weight on the plasma clearance of PEGylated dendrimers, which show a linear relationship between molecular weight and elimination half-life [18,40]. While the cleavable construct showed evidence of biodegradation in vivo (higher renal elimination coupled with evidence of lower molecular weight biodegradation products via SEC in urine), the extent of biodegradation observed over 3 days was evidently not high enough to significantly alter plasma clearance. While these renal elimination data are consistent with a recent study in healthy mice given 31 or 53 kDa PEG-based HBPs, the extent of renal excretion seen in mice was more varied, with 60% and 10–15% excretion of these polymers, respectively, over 5 h [41]. Another study with similar sized (48 kDa PEG-based HBP) bearing noncleavable EGDMA crosslinkers also showed shorter elimination half-lives of approx. 13.4 h in Balb/c nude mice bearing breast tumours, despite having a similar surface charge, molecular weight and polymeric composition with the HBPs explored here [23]. However, mice inherently exhibit more rapid plasma elimination of drugs and nanomaterials than rats [42] and immunocompromised animals (such as nude rodent models) also show more avid MPS uptake of dosed nanomaterials and, therefore, more rapid plasma clearance [43,44].

Two distinct SEC peaks were obtained in urine for non-cleavable HBPs (Figure 4A,B) suggesting the possibility of urinary clearance of the intact polymer as well as liberated ^89^Zr. Three possible mechanisms can explain the urinary clearance of the non-cleavable HBPs. Firstly, the z-average particle sizes of non-cleavable HBPs were <10 nm, which also include particles with population <6 nm, as evident by its high molar mass dispersity obtained from DLS measurements. These relatively small-sized populations of polymeric particles lower than the renal filtration threshold could have allowed excretion of the unchanged HBP via urine. Secondly, while EGDMA-based crosslinkers are relatively stable in the physiological environment compared to disulphide-based crosslinkers, the possibility of partial hydrolysis of the ester bond inherited from the methacrylate in EGDMA cannot be eliminated. This may result in the partial detachment of PEG chains from the intact polymers [45]. Thirdly, it is possible that a small portion of ^89^Zr may be incorporated non-specifically with the larger HBPs and was liberated in vivo and eliminated via the kidneys or deposited in bone (Figure 5). To this end, studies have suggested that the DFO-chelator complex is partly unstable in vivo, potentially allowing in vivo liberation of ^89^Zr followed by urinary elimination or bone deposition, which is a major target for many radiotracers, such as ^89^Zr [46,47]. Further, the intact ^89^Zr labelled polymers could be distributed in the bone without being liberated. Unfortunately, it was not possible to obtain an SEC analysis of radioactive material in bone marrow to confirm the time distribution trend without culling the rats.

In contrast to the lack of significant differences in the plasma pharmacokinetics of the HBPs, molecular weight and crosslinker chemistry affected the organ biodistribution of polymers. Specifically, the largest non-cleavable HBP showed more extensive uptake and accumulation of the dose in the MPS, while the cleavable HBP showed higher distribution towards the kidneys. In line with the biodistribution trend in our study, high molecular weight PEGylated-dendrimers (approximately 68 kDa) show maximum retention in MPS organs, while smaller molecular weight PEGylated dendrimers show limited MPS uptake and higher distribution to kidneys [18]. In agreement with our data for NC-HBP-22K, a recent biodistribution study that employed fluorescence imaging in healthy mice with slightly larger 28.5 kDa PEG-based HBPs demonstrated low uptake by the spleen and liver compared to other organs [48]. While this study provides a good comparison in terms of overall trend in organ distribution with NC-HBP-22K, the percentage of dose accumulated in each organ was not reported. A recent study of PEG-based HBPs employing an EGDMA crosslinker in tumour-bearing mice also demonstrated major deposition of polymers in highly vascularised tissues such as liver, spleen and heart with approximately 4, 3 and 3% injected dose/gram tissue, respectively at 3 days post-IV dose [23].

## 5. Conclusions

This study showed that PEG-based HBPs within the molecular weight range of 22–50 kDa show no clear differences in plasma pharmacokinetics, regardless of the presence or absence of cleavable linkers. Although renal elimination was expected to be efficient within this size range, the proportion of dose excreted via the urine was generally limited, with only up to 15% of the IV dose of the large cleavable HBP excreted over the first 3 days. In contrast, uptake by MPS organs was significantly affected by HBP size and crosslinker chemistry, whereby the higher MW non-degradable polymer accumulated over time in MPS organs, while MPS levels declined over time for the larger biodegradable HBP, and the smaller HBP due to continuing polymer degradation or equilibration with plasma concentrations. Nevertheless, these results suggest that the incorporation of biodegradable linkers into HBP structures can dictate patterns of biodistribution within, and clearance from the body, while the plasma pharmacokinetics of PEG-based HBPs are unaffected over the 20–50 kDa size range.

## Figures and Tables

**Table 1:** Physicochemical properties of hyperbranched polymers.

Polymers	Molecular Weight ^a^ (kDa)	Hydrodynamic Diameter (nm) (PDI) ^b^	Zeta Potential (mV)
NC-HBP-22K	22.3	6.3 (0.20)	0.86
NC-HBP-48K	48.2	9.0 (0.54)	−1.24
C-HBP-46K	45.5	8.2 (0.65)	−0.46

NC denotes non-cleavable ethylene glycol methacrylate (EGDMA)-based hyperbranched polymers (HBPs), while C denotes the cleavable disulphide HBP. ^a^ Molecular weight was determined via size exclusion chromatography (SEC) that uses a refractive index detector with linear polystyrene beads as control. ^b^ Particle size and polydispersity index measured by dynamic light scattering (DLS). Particle diameter is expressed as number-based distribution (*n* = 3).

**Table 2:** Pharmacokinetic parameters of HBPs after intravenous administration to rats at a dose of 15 mg/kg. Data are expressed as mean ± SD (*n* = 3 rats, except for NC-HBP-48K where *n* = 2 ± range).

Parameter	Unit	NC-HBP-22K	NC-HBP-48K	C-HBP-46K
K_el_	(h^−1^)	0.024± 0.001	0.027 ± 0.003	0.020 ± 0.001 *
t_1/2_	h	29.5 ± 0.8	25.8 ± 4.0	34.5 ± 2.3 *
Cp°	µg/mL	341 ± 7	384 ± 50	407 ± 52
Vc	mL	11.6 ± 0.9	11.2 ± 1.8	7.3 ± 0.7 ^#,^*
Vdb	mL	15.2 ± 0.4	15.9 ± 2.1	18.1 ± 5
AUC_0–72 h_	µg/mL.h	9045 ± 498	8646 ± 292	8236 ± 1839
AUC_0–inf_	µg/mL.h	11044 ± 589	9914 ± 22	9668 ± 633
Cl	mL/h	0.36 ± 0.002	0.43 ± 0.001	0.37 ± 0.001
AUC_0–72 h_	µg/mL.h	9045 ± 498	8646 ± 292	8236 ± 1839
AUC_0–inf_	µg/mL.h	11044 ± 589	9914 ± 22	9668 ± 633
Dose excreted in urine	%	6.3 ± 2.3	9.3 ± 5.3	14.5 ± 0.9 ^#^
Dose excreted in feces	%	3.7 ± 0.2	2.4 ± 0.1	9.9 ± 1.1 ^#,^*

Statistical comparisons were undertaken via one-way ANOVA followed by Tukey’s test for least significant differences (*p* ≤ 0.05). ^#^ indicates *p* < 0.05 compared with NC-HBP-22K; * indicates *p* < 0.05 compared with NC-HBP-48K.

## Supplementary Materials

The following are available online at https://www.mdpi.com/2079-4991/10/12/2452/s1, Methods for characterisation of polymers via size exclusion chromatography, nuclear magnetic response (NMR) spectroscopy and particle size and zeta potential measurements. Table S1: Materials used in the preparation of hyperbranched polymers, Figure S1: NMR spectra of A) NC-HBP-22K B) NC-HBP-48K after and C) C-HBP-46K after DFO (chelator) conjugation. The peak at ~ 3.33 ppm was assigned to water. Figure S2: Organ biodistribution of ^89^Zr-labelled individual HBPs at 4, 24 and 72h post-injection showing accumulation of polymers. Region of interest (ROI) analysis of whole body and organs allow determination of in vivo distribution of HBPs in the organs. Biodistribution across organ at various timepoints were calculated by ROI analysis of PET/CT images. Images are decay-corrected to the point of dosing and intensity at each organ are expressed as % ID/g of tissue (*n* = 3 rats for all groups except NC-HBP-48K where *n* = 2 rats and data are represented as mean ± range). Statistical significance was conducted using one-way ANOVA followed by Tukey’s test. * indicates *p* < 0.05 compared with NC-HBP-22K.
