# Supplementary material for: The Impact of Polymer Size and Cleavability on the Intravenous Pharmacokinetics of PEG-Based Hyperbranched Polymers in Rats
Source: Nanomaterials (Basel). 2020 Dec 8;10(12):2452. doi: 10.3390/nano10122452 (PMC7762536; doi:10.3390/nano10122452)
Supplement: Supplementary file 1 [file nanomaterials-10-02452-s001.pdf]

nanomaterials

Supporting information

The Impact of Polymer Size and Cleavability on The Intravenous Pharmacokinetics of PEG-Based Hyperbranched Polymers in Rats

Nirmal Marasini [1,*], Changkui Fu [2], Nicholas L. Fletcher [2,3,4], Christopher Subasic [1], Gerald Er [2], Karine Mardon [3], Kristofer J. Thurecht [2,3,4], Andrew K. Whittaker [2] and Lisa M. Kaminskas [1,*]

1 School of Biomedical Sciences, The University of Queensland, St Lucia 4072, Queensland, Australia; christopher.subasic@uq.net.au
2 Australian Institute for Bioengineering and Nanotechnology, The University of Queensland, St Lucia 4072, Queensland, Australia; changkui.fu@uq.edu.au (C.F.); n.fletcher1@uq.edu.au (N.L.F.); tze.er@uq.net.au (G.E.); k.thurecht@uq.edu.au (K.J.T.); a.whittaker@uq.edu.au (A.K.W.)
3 ARC Centre of Excellence in Convergent Bio-Nano Science and Technology, The University of Queensland, St Lucia 4072, Queensland, Australia
4 ARC Training Centre for innovation in Biomedical Imaging Technology, The University of Queensland, St Lucia 4072, Queensland, Australia
5 Centre for Advance Imaging, The University of Queensland, St Lucia 4072, Queensland, Australia; k.mardon@uq.edu.au
* Correspondence: n.marasini@uq.edu.au (N.M.); l.kaminskas@uq.edu.au (L.M.K.)

Characterisation

Size Exclusion Chromatography (SEC)

HBP molecular weight and the distribution patterns were determined using a Polymer Laboratories GPC50 plus equipped with a differential refractive index detector, dual angle light scattering detector, and viscometer. Chromatograms were collected using two PLGel Mixed B (7.8 × 300 mm) SEC columns connected in series and held at a constant temperature of 50 °C. Samples (10-15 mg/mL) were passed through 0.45 µm PTFE syringe filter and eluted at a flow rate of 1.0 mL/min using HPLC grade *N*,*N*-dimethylacetamide (DMAc, containing 0.03 wt % LiCl) solvent. The system was calibrated using polystyrene standards with molecular weights ranging from 6.82×10^2 g/mol to 1.67×10^6 g/mol.

Nuclear Magnetic Resonance (NMR)

Polymers were dissolved in DMSO-d6, and ^{1}H NMR spectra were obtained using a Bruker Avance 400 MHz spectrometer at 298 K (Bruker, USA).. All chemical shifts are reported in ppm (δ) relative to tetramethylsilane (TMS).

Particle size and zeta potential measurements

HBPs (10 mg/mL) were dispersed in Milli Q water for size analysis. The size of resultant nanoparticles were determined by dynamic light scattering (DLS) particle size analyser (Zetasizer Nano series ZS, Malvern Instruments, Malvern, UK) at a scattering angle of 173° and 25°C. At least triplicate measurements determined the mean size of particles at room temperature and the values of z-average diameters were used. The polydispersity index (PDI) of the nanoparticles were also determined. Zeta potentials were measured using folded capillary zeta cells at a similar concentration (10 mg/mL) in milliQ water. At least three replicate experiments were conducted and average measurements were used.

Table S1. Recipes used in the preparation of hyperbranched polymers.

	**NC-HBP-22K Molar Ratio (1:4:42:1.5:0.2)**	**NC-HBP-48K Molar Ratio (1:4:128:1.5:0.2)**	**C-HBP-46K Molar Ratio (1:4:128:1.5:0.2)**
CTA (4-Cyano-4-[(dodecylsulfanylthiocarbonyl)sulfanyl]pentanol	19 mg	9.5 mg	9.5 mg
Ethylene glycol dimethacrylate	40 mg	20 mg	
N,N′-Bis(acryloyl)cystamine (BAC)			26 mg
Poly (ethylene glycol) methyl ether acrylate (PEGMA)	1000 mg	1500 mg	1500 mg
2-aminoethyl methacrylate hydrochloride	12 mg	6 mg	6 mg
AIBN Azobis (isobutyronitrile)	1.57 mg	0.8 mg	0.8 mg

A)

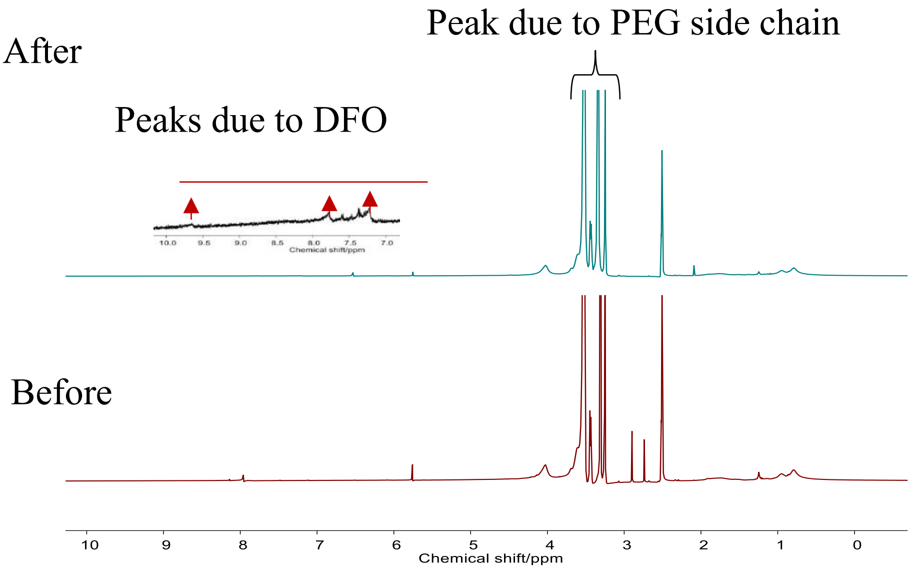
After
Peak due to PEG side chain
Peaks due to DFO
Before
10
9
8
7
6
5
4
3
2
1
0
Chemical shift/ppm

B)

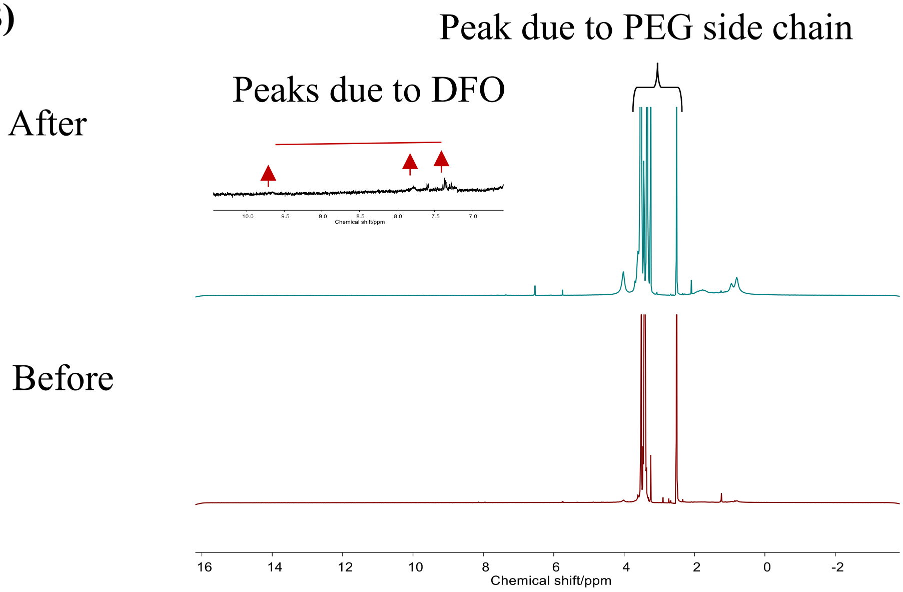
Peak due to PEG side chain
Peaks due to DFO
After
Before
16
14
12
10
8
6
4
2
0
-2
Chemical shift/ppm

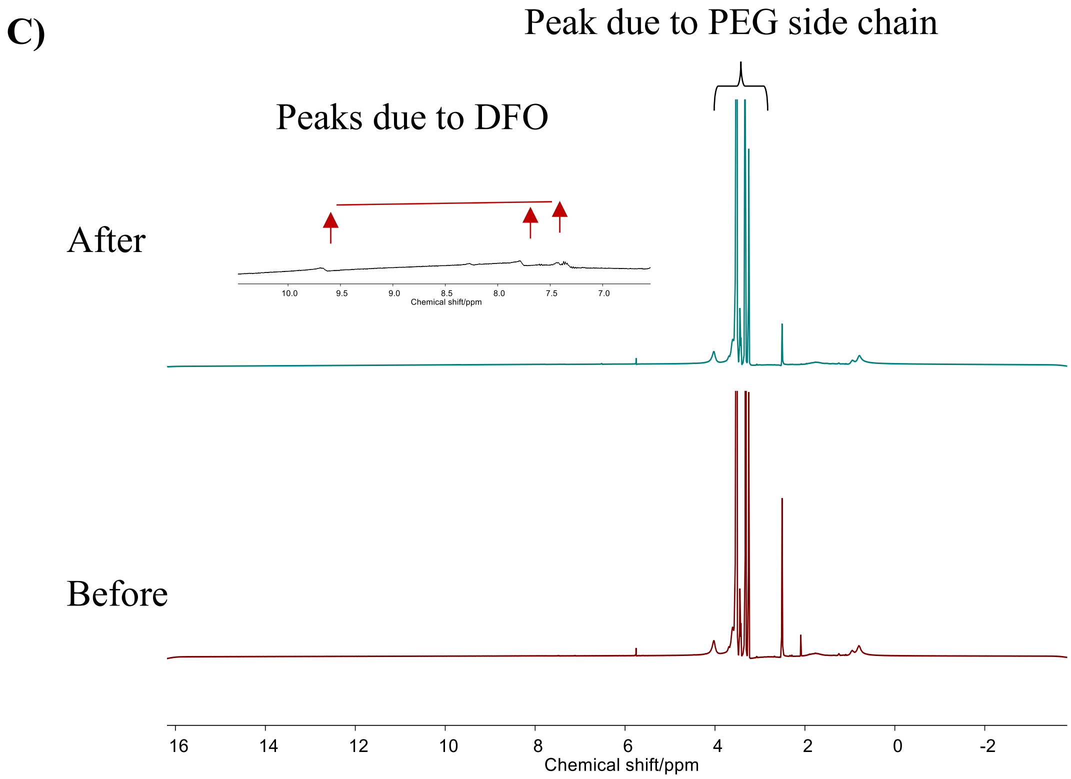

Figure S1. NMR spectra of **A**) NC-HBP-22K **B**) NC-HBP-48K after and **C**) C-HBP-46K after DFO (chelator) conjugation. The peak at ~ 3.33 ppm was assigned to water.

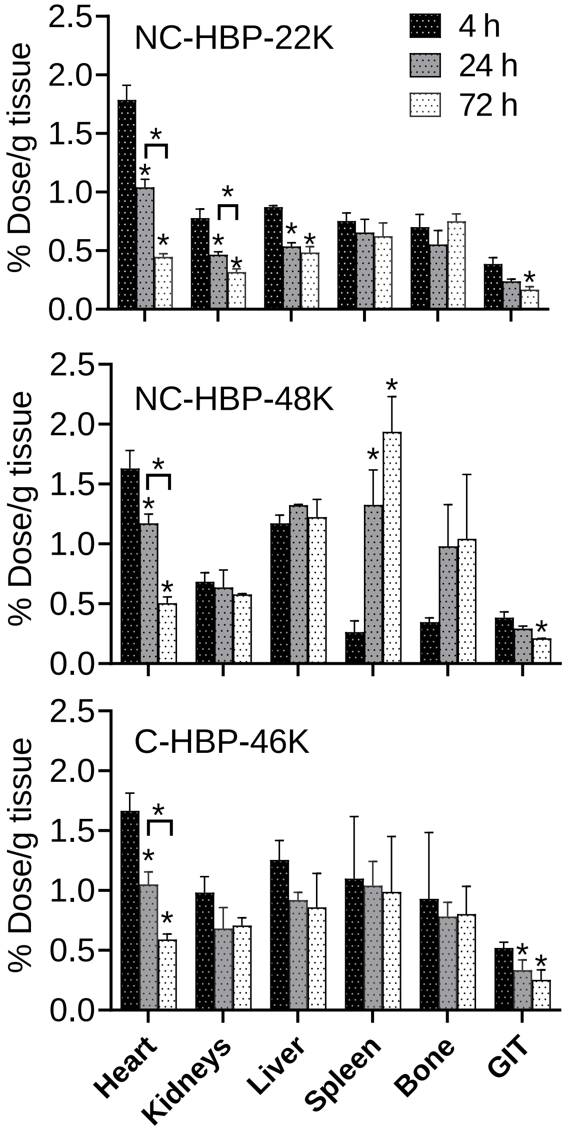

Figure S2. Organ biodistribution of ^{89}Zr labelled individual HBPs at 4h, 24h and 72h post-injection showing accumulation of polymers. Region of interest (ROI) analysis of whole body and organs allow determination of in vivo distribution of HBPs in the organs. Biodistribution across organ at various time points were calculated by ROI analysis of PET/CT images. Images are decay corrected to the point of dosing and intensity at each organ are expressed as % ID/g of tissue (n = 3 rats for all groups except NC-HBP-48K where n = 2 rats and data are represented as mean ± range). Statistical significance was conducted using one-way ANOVA followed by Tukey's test. * indicates $p < 0.05$ compared with NC-HBP-22K.
